# Acute limb ischemia caused by floating thrombus in the aorta: a case report and literature review

**DOI:** 10.3389/fcvm.2023.1203003

**Published:** 2023-06-28

**Authors:** Fuzheng Guo, Zhibin He

**Affiliations:** ^1^ Trauma Center, National Center for Trauma Medicine, Key Laboratory of Trauma and Neural Regeneration (Ministry of Education), Peking University People’s Hospital, Beijing, China; ^2^ Department of Vascular Surgery, Peking University People's Hospital, Beijing, China

**Keywords:** aortic arch, floating thrombus, embolism, atherosclerosis, COVID-19

## Abstract

This report presents a patient with rheumatoid arthritis and COVID-19 infection one month earlier who experienced embolic episodes resulting in acute lower-limb ischemia from an unusual source. The blood flow was successfully restored by femoropopliteal thromboembolectomy. In determining the source of the embolism, the patient underwent electrocardiogram, transthoracic echocardiogram, and aortic CTA. The latter revealed a large, pedunculated, and mobile thrombus arising from the aortic arch and the descending thoracic aorta. Considering the patient's general health condition, we performed anticoagulation of the floating thrombus in the aortic lumen. The mechanism of aortic floating thrombosis exhibits considerable complexity. There are no standardized treatment protocols or clinical guidelines, and its treatment mainly includes open surgery, aortic endoluminal stent -graft insertion and pharmacological anticoagulation. Treatment strategy should be based on the cause of the disease and the patient's physical condition.

## Introduction

With China's deregulation of COVID-19 epidemic control, there is increasing evidence of the nonrespiratory effects of the coronavirus, including the development of coagulopathy-related manifestations such as arterial thromboembolism. The above has increased the vigilance of vascular surgeons. Multiple risk factors are associated with the formation and development of arterial thrombosis. Age, sex, history of coronary artery disease, and prior myocardial infarction are associated with thrombotic events following COVID-19 infection (1). Most arterial thromboses are acute myocardial infarction or ischemic stroke (2). Reports of aortic thrombosis are less frequent in cases of arterial thrombosis complications (3). Stroke and peripheral arterial embolism are commonly suspected to originate from the heart. Because of the large diameter of the aorta, high flow, and absence of significant mural atherosclerosis, thrombosis is difficult to form and persist.

With the increasing use of imaging techniques in recent years, floating aortic thrombi have become increasingly recognized as systemic emboli, creating a life-threatening risk of ischemic stroke or peripheral emboli. The pathophysiological mechanisms underlying this disease are not fully understood (4). Several authors have reported mobile thrombi arising from aneurysms and protruding atheromas that are significantly related to aging (5). However, in patients without severe atherosclerosis, floating aortic thrombi are also observed. We report a case of a pedunculated thrombus within the aorta, resulting in acute lower limb ischemia.

## Case description

**Figure 1:**
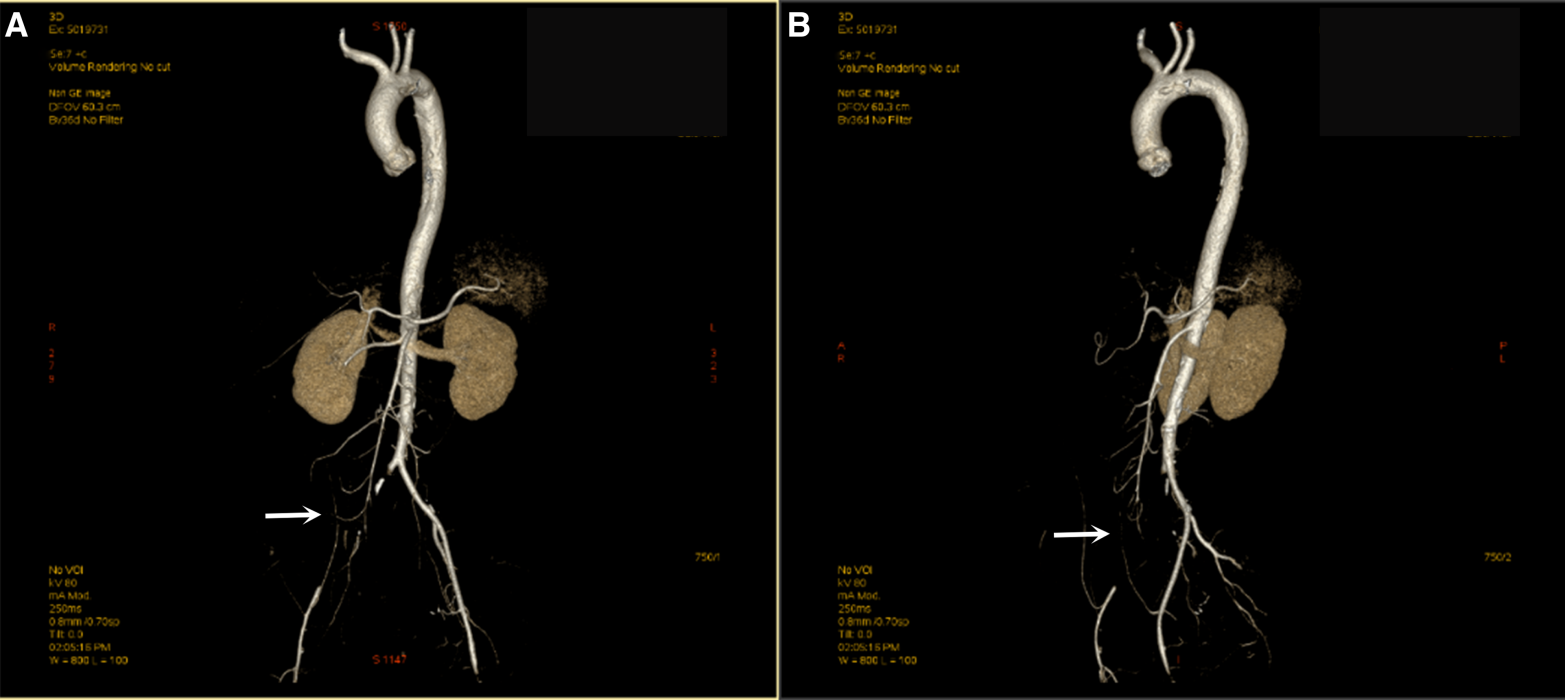
Computed tomography angiography (CTA) shows embolization of the right common iliac artery. The right common iliac artery was not filled with contrast, and the distal vessels were slimmer than the left (**A,B**).

**Figure 2:**
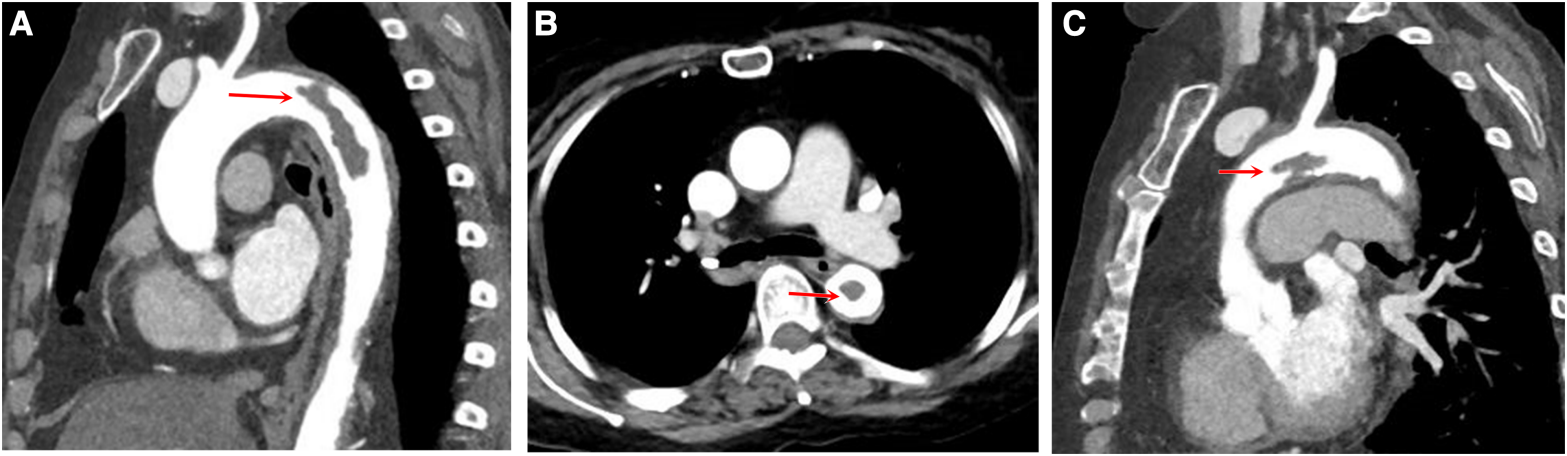
Aortic computed tomography scan displaying floating thrombus in the aortic lumen. The multiplanar reconstruction of CTA shows a filling defect in the descending aorta (**A**) and in the aortic arch (**C**), with the long axis in line with the direction of blood flow, proximally attached to the wall and distally free. The cross-sectional view shows the floating thrombus in the descending aorta (**B**).

A 62-year-old woman was admitted with acute pain, coldness, and anesthesia in the right lower limb. No palpable pulses were observed in the popliteal, posterior tibial, or dorsalis pedis. The pulses in the left lower limb were normal. The patient had a history of rheumatoid arthritis (RA) but did not receive regular treatment. She had a history of cigarette smoking (36 pack/years). One month ago, the patient was infected with COVID-19 and was treated at home due to mild symptoms. The result of patient's nucleic acid test for COVID-19 was negative on admission. The patient received serological analysis to detect hypercoagulable and vasculitis disorders, including antiphospholipid antibodies. Laboratory test results showed no apparent abnormalities. Computed tomographic angiography (CTA) of the limb revealed no contrast agent over the entire course of the right common iliac artery, external iliac artery, or proximal segment of the internal iliac artery (Figure 1). Electrocardiography revealed sinus tachycardia (Supplementary Figures S1, S2). Echocardiography and cervical artery duplex ultrasonography were non-contributory. The chest x-ray was normal, and aortic CTA was performed before surgery, demonstrating a large filling defect caused by a floating thrombus in the aortic arch and the descending thoracic aorta (Figure 2, Supplementary Figure S3).

**Figure 3:**
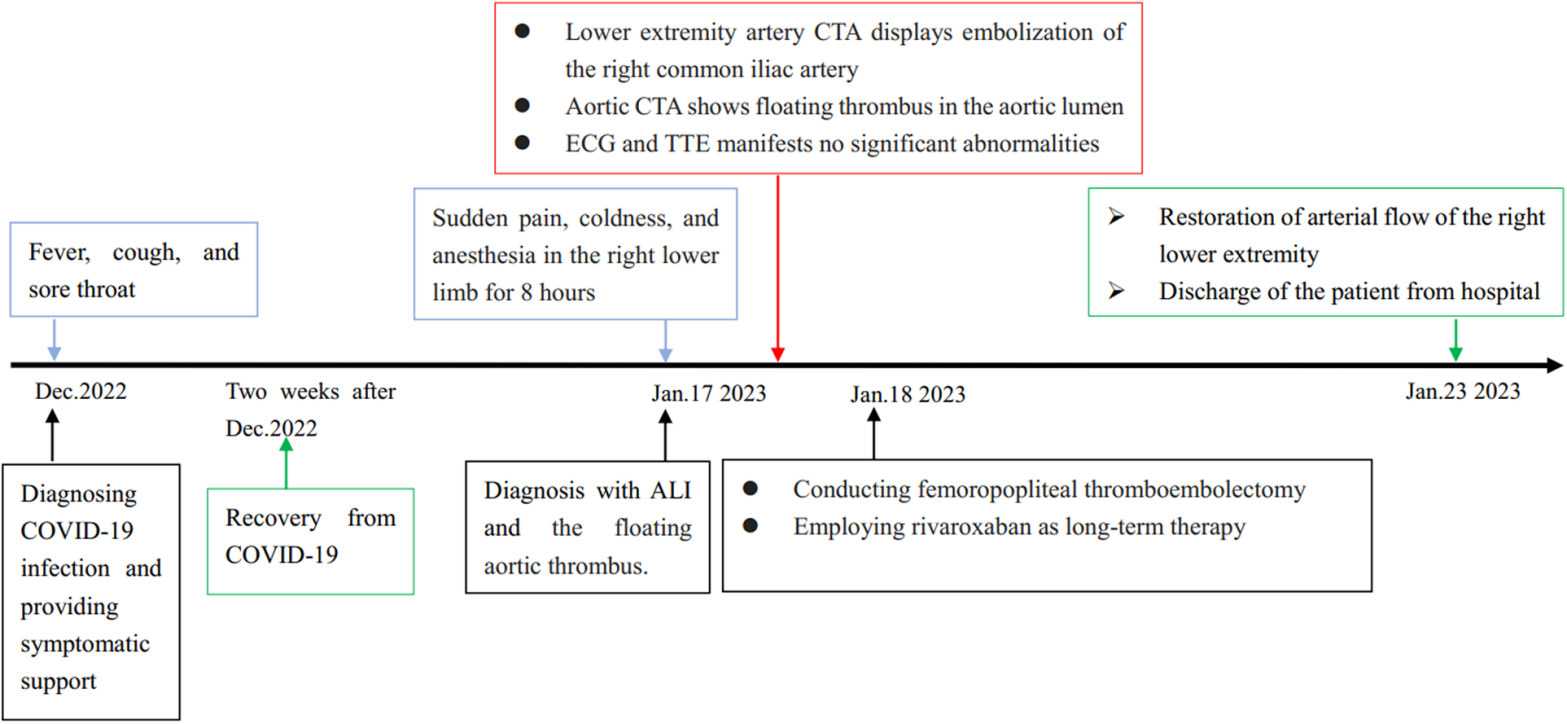
Timeline.

The diagnosis of acute lower extremity ischemia due to a floating aortic thrombus was clear based on the patient's symptoms, signs, and imaging data. Figure 3 was showcasing a timeline with relevant data from the episode of care. A femoropopliteal thromboembolectomy was performed, which removed the embolus and restored limb perfusion (Supplementary Figure S4). After surgery, she received low-molecular-weight heparin treatment (Enoxaparin, 6000I U/12 h). We administered adequate anticoagulation using rivaroxaban at a daily dosage of 20 mg to prevent thrombotic progression post-discharge. Additionally, cilostazol, a phosphodiesterase inhibitor, was prescribed to enhance blood supply to the lower extremities and prevent platelet aggregation. Concurrently, patients were advised to stop smoking. The patient was apprised of the potential for thrombus re-dislodgement and advised to undergo anticoagulation therapy for a minimum of 3–6 months, with subsequent treatment contingent upon the management of rheumatoid arthritis.

## Discussion

Patients with severe COVID-19 infections may experience arterial thrombotic events such as acute coronary infarction, stroke, and limb ischemia (1). Although aortic thrombosis is uncommon, it has been reported in COVID-19 patients (3), and its pathophysiology is not fully defined. A procoagulant nature may develop aortic thrombosis due to various diseases, including sepsis, disseminated intravascular coagulation (DIC), autoimmune diseases, pregnancy, and cancer. The most common location was the descending thoracic aorta and the aortic arch (6). Protruding atheromas and aneurysms seem to be closely associated with the thrombosis in aorta (7). However, the pathogenic mechanism was unsuitable for younger patients because the aortic wall of these patients was normal or the atherosclerosis was not severe. Consequently, there may be a separate clinical entity from atheromatous disease in relatively young individuals. An adherent thrombus in the non-atherosclerotic aortic wall could represent an endothelial arterial disorder. Many studies have revealed that steroids can damage the endothelium directly and cause thickening of the intima (8). Oral contraceptives or pregnancy have been reported to be closely associated with intimal hyperplasia (9). Also, it has been reported that viral infections are related to thrombotic events (3). Inflammation, endothelial cell injury, platelet activation, and hypercoagulability contribute to viruses associated coagulopathy.

In this case, we believe that the floating aortic thrombus was caused by multiple factors.

First, although the patient denied a history of cardiovascular disease, she was at a high risk of atherosclerotic cardiovascular disease because of tobacco use, elevated blood pressure, and obesity (10). Second, the patient had a history of rheumatoid arthritis and oral steroid hormones. Studies have pointed out that patients with rheumatoid arthritis have an increased risk of cardiovascular disease compared to the general population, with a 68% higher risk of myocardial infarction and a 41% higher risk of stroke (11). Because of premature atherosclerotic development, high endothelial dysfunction rates, and rapid progression of atherosclerosis, RA patients are more likely to have cardiovascular events (12). It is accepted that atherosclerosis is a chronic inflammatory disease like RA. There are significant similarities between these two diseases in terms of their pathogenic and genetic factors. Inflammation associated with rheumatoid arthritis increases the incidence of vascular insults and the progression of atherosclerosis (13). Glucocorticoids can effectively suppress RA-related inflammation. However, there are numerous adverse cardiovascular effects associated with their use, including stroke, myocardial infarction, and heart failure. The adverse effects on lipid metabolism may account in part for their detrimental cardiovascular effects (11). Third, the patient was infected with COVID-19 one month ago. Similar to other coronaviruses, a significant association has been found between COVID-19 and the risk of thrombosis. A meta-analysis found that arterial thrombosis occurs in 4.4% of cases (14). The pathophysiology of thromboembolism in COVID-19 involves several aspects. Thrombotic and thromboembolic events are predisposed to COVID-19 because of excessive inflammation, endothelial injury, platelet activation, and hypercoagulability in the blood.

No standard protocol is available for treating floating aortic thrombi (15). Treatment options include anticoagulation, surgical thrombectomy, and endovascular treatment (7). Under comprehensive consideration, anticoagulation therapy was selected for aortic floating thrombus.

## Conclusion

In the context of the novel coronavirus pandemic, COVID-19 could predispose patients to both venous and arterial thrombotic events. Although arterial thrombosis is a rare complication of this disease compared to venous thromboembolism, it cannot be underestimated. Aortic thrombosis is infrequent in arterial thrombosis, and some cases are diagnosed during screening for other diseases. Larger case series studies are required to determine whether aortic thrombosis is an uncommon complication of thrombotic events linked to COVID-19. Meanwhile, the procoagulant nature of RA is correlated with thrombosis in atypical locations and should be considered in patients with cardiovascular events. Evaluating the aorta is warranted in all patients with peripheral emboli of uncertain pathogenesis.

## Data Availability

The original contributions presented in the study are included in the article/**Supplementary Material**, further inquiries can be directed to the corresponding author.

## Supplementary material

Supplementary Figure 1 The patient's bedside electrocardiogram at the time of admission. ECG results showed sinus rhythm with a heart rate of 112 beats per minute.

Supplementary Figure 2 ECG repeated the next day manifests a heart rate of 91 beats per minute with sinus rhythm.

Supplementary Figure 3 Revascularization imaging of aortic CTA; thick arrows indicate floating thrombus.

Supplementary Figure 4 (**A**) Dislodged thrombus in the iliac artery. (**B**) The femoral artery is dissected out, and the proximal and distal ends are banded separately.
