# Supplementary figures and images for: Acute limb ischemia caused by floating thrombus in the aorta: a case report and literature review
Source: Front Cardiovasc Med. 2023 Jun 28;10:1203003. doi: 10.3389/fcvm.2023.1203003 (PMC10337781; doi:10.3389/fcvm.2023.1203003)

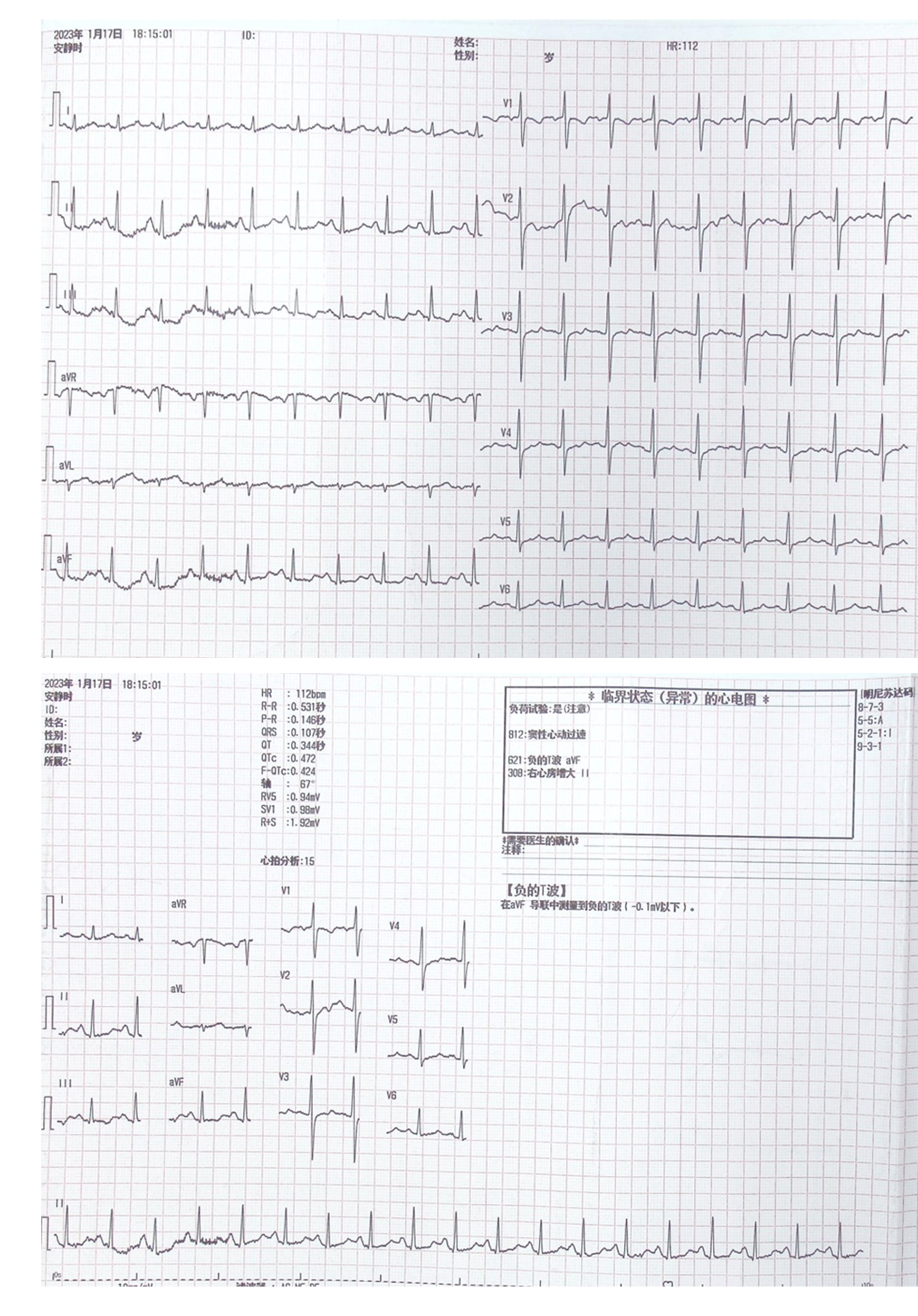

Supplement: Supplementary Figure 1 — The patient's bedside electrocardiogram at the time of admission. ECG results showed sinus rhythm with a heart rate of 112 beats per minute. [file Image1.tif]

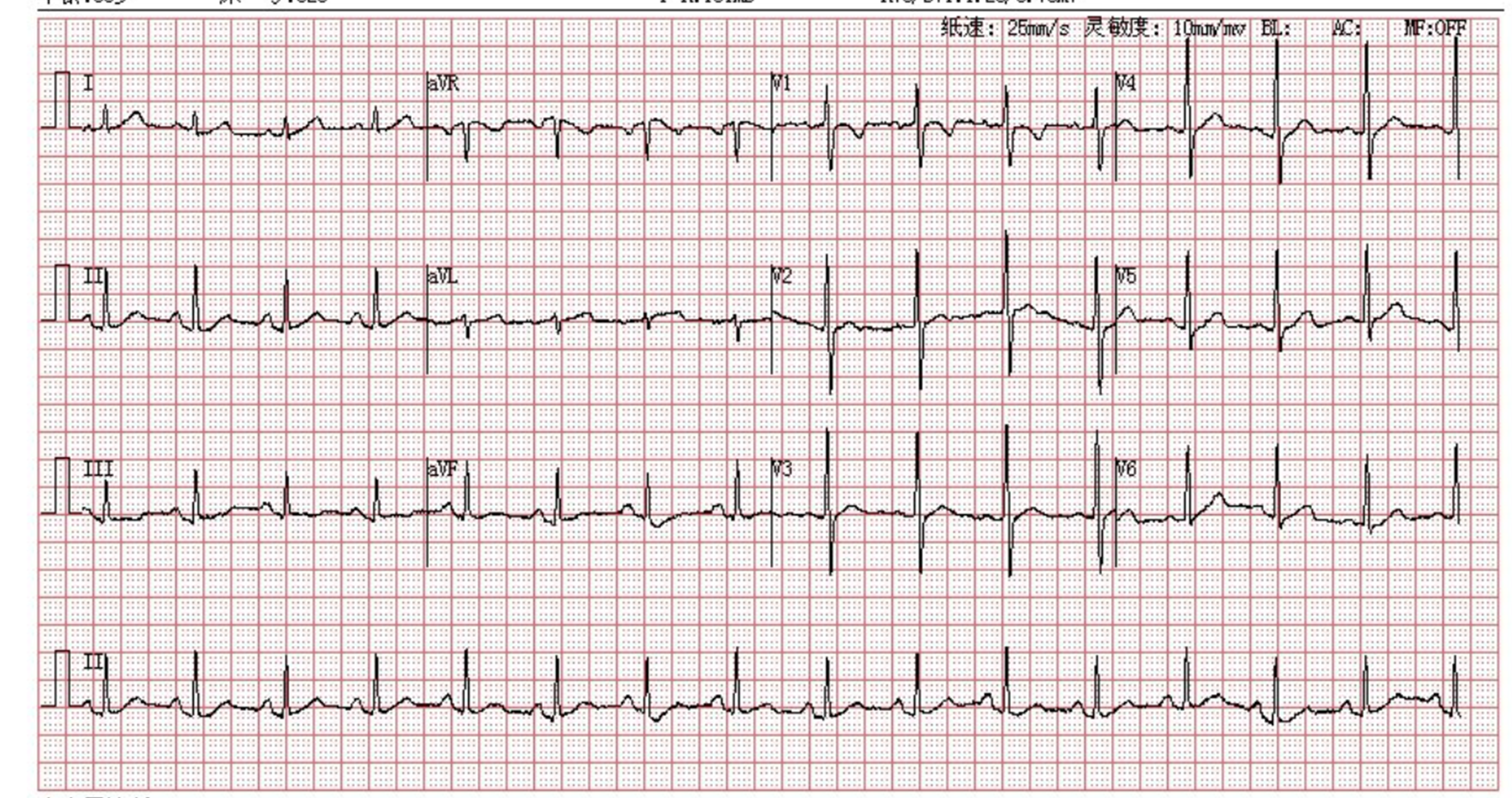

Supplement: Supplementary Figure 2 — ECG repeated the next day manifests a heart rate of 91 beats per minute with sinus rhythm. [file Image2.tif]

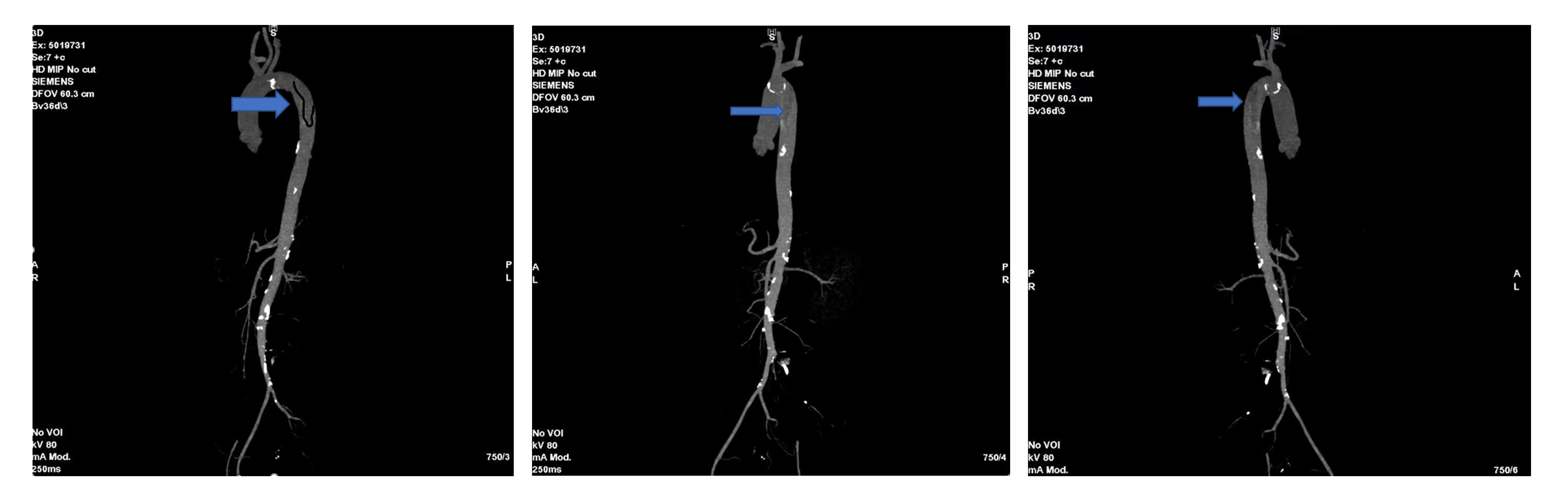

Supplement: Supplementary Figure 3 — Revascularization imaging of aortic CTA; thick arrows indicate floating thrombus. [file Image3.tif]

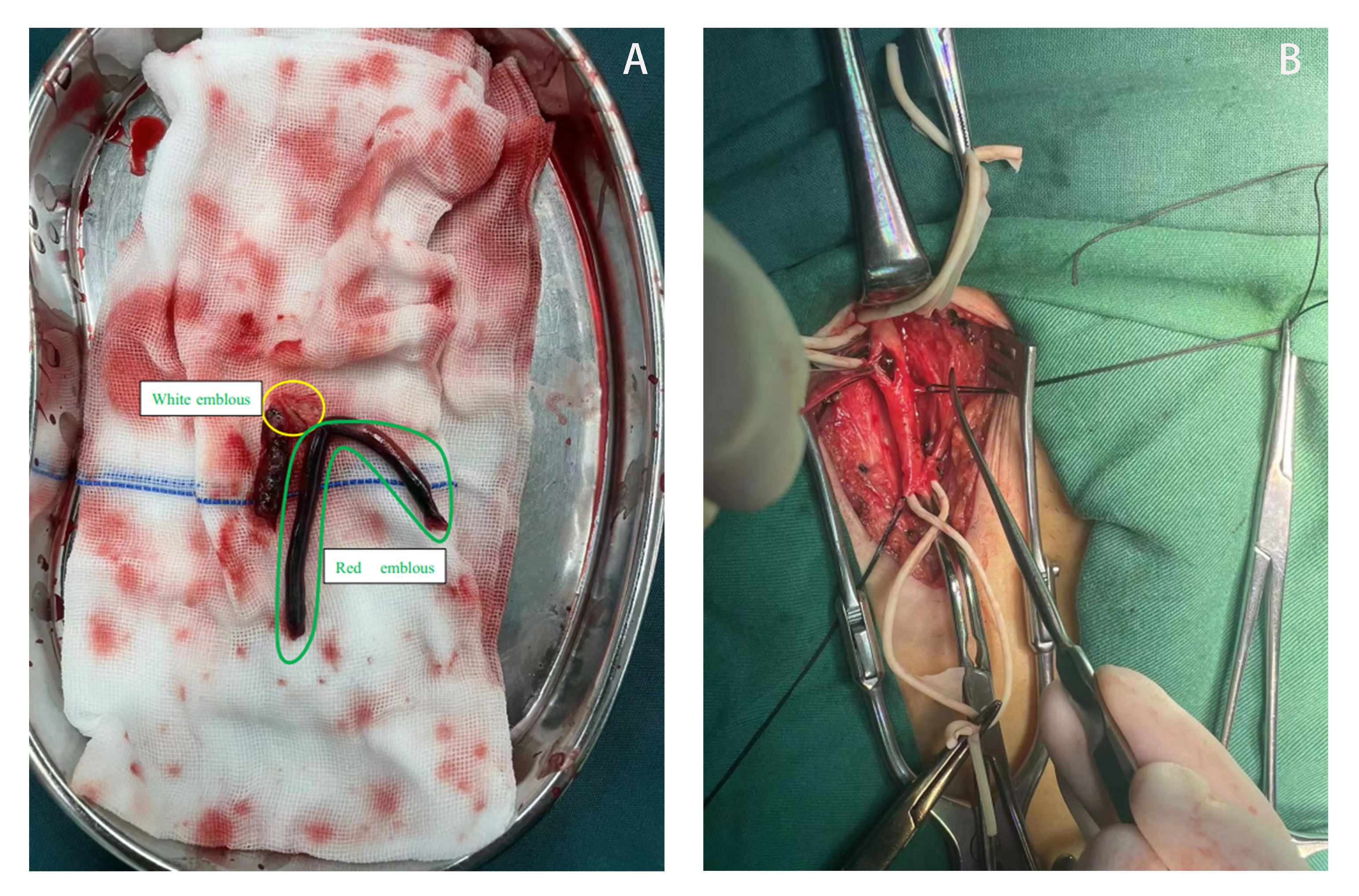

Supplement: Supplementary Figure 4 — (A) Dislodged thrombus in the iliac artery. (B) The femoral artery is dissected out, and the proximal and distal ends are banded separately. [file Image4.tif]
